# Sequencing of complete mitochondrial genome of brown algal *Saccharina* sp. ye-C5

**DOI:** 10.1080/23802359.2015.1137799

**Published:** 2016-02-05

**Authors:** Xiao Fan, Shuai Wang, Le Xu, Dong Xu, Xiaowen Zhang, Naihao Ye

**Affiliations:** a Yellow Sea Fisheries Research Institute, Chinese Academy of Fishery Sciences, Qingdao, China;; b Function Laboratory for Marine Fisheries Science and Food Production Processes, Qingdao National Laboratory for for Marine Science and Technology, China

**Keywords:** Complete mitochondrial genome, illumina sequencing, *Saccharina* sp. ye-C5

## Abstract

The complete genome (37 635 bp) mitochondrial DNA (mtDNA) of the *Saccharina* sp. ye-C5 was determined, which contains 38 protein-coding genes (PCG), three ribosomal RNA (rRNA) and 25 transfer RNA (tRNA) genes that are consistent in *Saccharina* genus. The phylogenetic tree that was established based on the mitochondrial genomes of brown algae, which indicated that *Saccharina* sp. ye-C5 and *Saccharina longissima* are the most closely related species.

Seaweeds are essential for marine ecosystems and have immense economic value. The kelp *Saccharina* (Phaeophyceae) is included decades ago and become an important economic industry (Wang et al. 2013). Recently, the whole genome of *Saccharina* has been uncovered (Ye et al. 2015), which provide new sights to the biology and evolution of *Saccharina.* Illumine sequencing data generated in the genome project have produced several mitochondrial genomes of different strains of *Saccharina* (Guan et al. 2014; Wang et al. 2014, 2015). Which may play an important role in the population genetic sand molecular evolution (Fan et al. 2012; Yuan et al. 2012). In this study, the complete mitochondrial genome of a wild strain, sampled in Japan (43°31′ N, 145°74′3°) and stored in Yellow Sea Fisheries Research Institute Algae Culture Center, Qingdao, China (numbered as YSFRI-Saccharina-sp-ye-C5), is uncovered and named as *Saccharina sp*. ye-C5 based on the phylogenetic analysis (NCBI accession number no. KT336421). The assembly and the annotation of mitochondrion genome were processed according to Wang et al. (2015).

The length of complete of *Saccharina* sp. ye-C5 is 37 635 bp and the genome contains 38 protein-coding genes (*rps2-4*, *rps7-8*, *rps10-14*, *rps19, atp6*, *atp8*, *atp9*, *cox1-3*, *nad1-7*, *nad9*, *nad11*, *nad4L*, *rpl2*, *rpl5*, *rpl6*, *rpl14*, *rpl16*, *rpl31*, *ORF41*, *ORF130*, *ORF377*, *tatC* and *cob*), 25 transfer RNA (tRNA) genes, three ribosomal RNA (rRNA) genes (*5S* rRNA, *16S* rRNA and *23S* rRNA). All 38 protein-coding genes (PCGs) have typical initiation codons (ATG). The numbers of PCGs that have complete termination codons TAA, TAG, TGA are 26, eight and four, respectively. The overall GC content is 35.34%, which is well within the normal range of brown mitochondrial DNAs. Nucleotide frequency of the H-strand is as follows: T, 36.25% A, 28.41%; C, 14.74%; and G, 20.60%. The mitogenome of *Saccharina* sp. ye-C5 encodes 9637 amino acids, excluding the stop codons. All the 25 typical tRNAs, ranging from 71 to 88, possess a complete clover leaf secondary structure. The rRNAs of the *5S* rRNA, *16S* rRNA and *23S* rRNA genes are 133 bp, 1535 bp and 2745 bp in length, respectively.

In this study, we found that *Saccharina* sp. ye-C5 belongs to a *Saccharina* clade and are closely related to *S. longissima.* This phylogenetic analysis integrated other 16 brown algae complete mitochondrial sequence data forcefully agreed with the former phylogenetic analyses (Zhang et al. 2013; Guan et al. 2014). Complete mitochondrial genomes have enhanced the resolution and statistical confidence of inferred phylogenetic trees (Figure 1).

**Figure 1.**
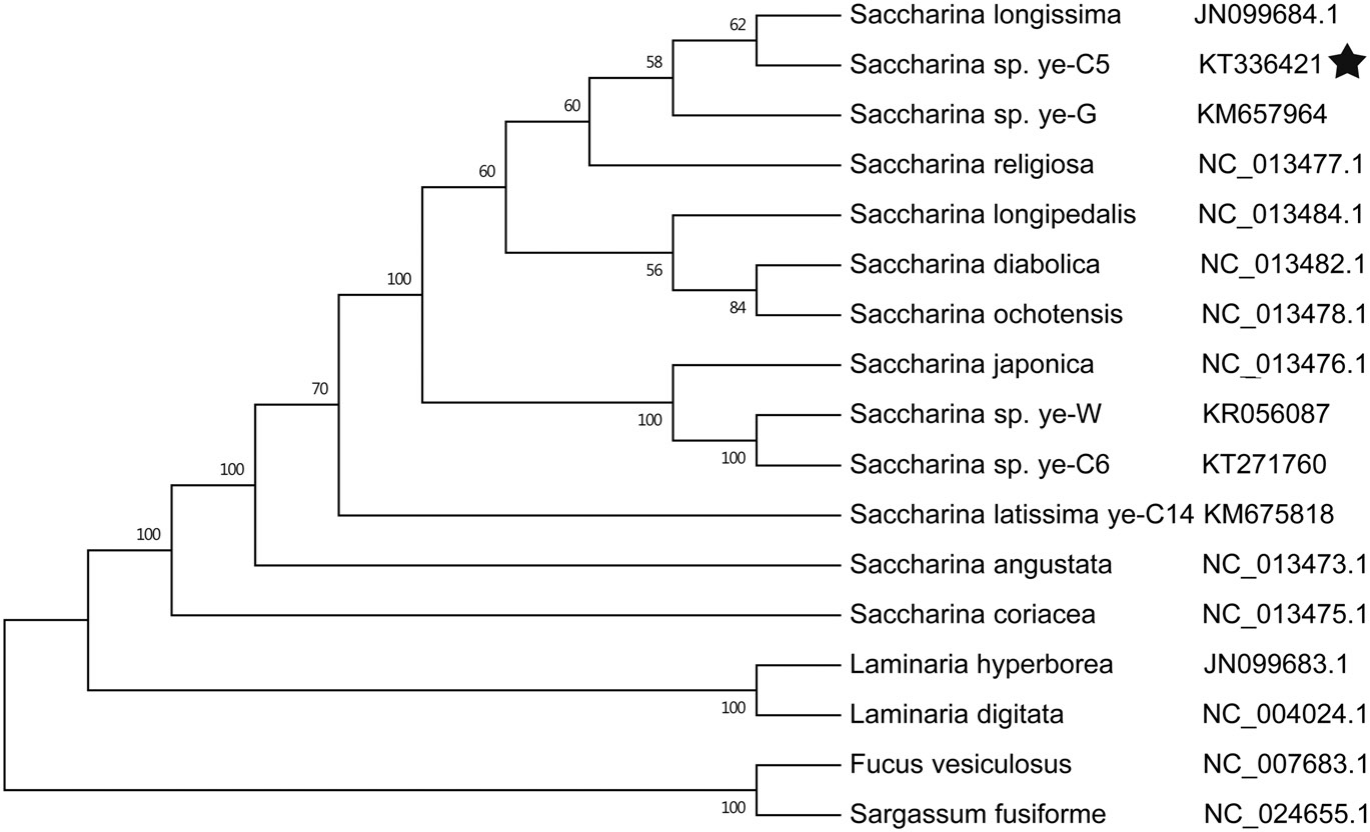
Phylogenetic tree of ML analyses based on the complete mitochondrial nucleotide acid sequences of other brown algae. The pentagram stands for the new sequenced species in our work
